# Locked vs. unlocked AED cabinets: The Western Australian perspective on improving accessibility and outcomes

**DOI:** 10.1016/j.resplu.2024.100807

**Published:** 2024-12-19

**Authors:** Matthew Didcoe, Caitlyn Pavey-Smith, Judith Finn, Jason Belcher

**Affiliations:** a St John WA, Western Australia, Australia; b Prehospital Resuscitation and Emergency Care Research Unit, Curtin University, Western Australia, Australia

To the Editor

We appreciate the work of Oonyu et al. on behalf of the ILCOR BLS Task Force in their recent paper, “The impact of locked cabinets for automated external defibrillators (AEDs) on cardiac arrest and AED outcomes: A scoping review”^1^ and agree that further research is needed in this space to improve cardiac arrest outcomes.

We offer the Western Australian (WA) experience to illustrate how an integrated system can achieve positive outcomes even with locked cabinets. St John WA, the jurisdictional ambulance service covering 2.5 million square kilometres, has operated the State Defibrillator Network for over 13 years, with more than 10,000 registered AEDs − 28 % of which are available 24/7 in combination-lock cabinets. In 2023, 16 % of all non-EMS-witnessed OHCA in WA with an EMS resuscitation attempt had pads applied from a community AED before EMS arrival, rising to 43 % in public places. We intend to publish further details on our defibrillator network, but, based on our experience, we wish to provide commentary on the locked versus unlocked cabinet debate.

The debate is not simply “locked vs unlocked” − there are different types of locked cabinets. The literature in the review^1^ often describes cabinets with a small break-glass feature and key access. The Singaporean study^2^ reported injuries to rescuers using such a design. In WA, we use keypad combination locks, with the code provided during the emergency call. An integrated network allows an emergency call to activate both EMS and community first responders, and direct callers to the nearest AED, with access code information held in the ambulance computer-aided dispatch (CAD) system. This locked cabinet model has additional benefits beyond deterring vandalism and theft. A telephone call to obtain the unlock code ensures EMS has been activated, and recording the deployment in CAD facilitates prompt AED consumable replacement to maintain community availability.

Though the limited published data identified in the review suggests low rates of theft^1^, and one paper described some stolen AEDs being recovered through tracking devices,^3^ theft can cause temporary loss of PAD availability if recovered and financial burden if not recovered. Given that AEDs in many parts of the world including Australia are often funded by private organisations rather than government, even a single theft can be a deterrent to making AEDs publicly accessible. In WA, we have seen organisations removing PADs after incidents of vandalism or theft.

Our experience is that combination-locked cabinets are generally more acceptable to organisations considering making AEDs publicly accessible based on perception of risk. If unlocked cabinets are unacceptable in the view of organisations who invest in AEDs, the real debate might be “locked vs no PAD” rather than “locked vs unlocked.” Thus, understanding the acceptability of different cabinet types for PAD providers is essential, and we suggest this as a priority for further research. If locked cabinets encourage more 24/7 availability, research into optimal cabinet design and integration with emergency networks should be prioritised to improve OHCA patient outcomes.

## Declaration of competing interest

The authors declare that they have no known competing financial interests or personal relationships that could have appeared to influence the work reported in this paper.
